# Electric-Field-Assisted Synthesis of Cu/MoS_2_ Nanostructures for Efficient Hydrogen Evolution Reaction

**DOI:** 10.3390/mi15040495

**Published:** 2024-04-03

**Authors:** Surra Yonas, Birhanu Bayissa Gicha, Samir Adhikari, Fedlu Kedir Sabir, Van Tan Tran, Njemuwa Nwaji, Bedasa Abdisa Gonfa, Lemma Teshome Tufa

**Affiliations:** 1 Department of Applied Chemistry, Adama Science and Technology University, Adama P.O. Box 1888, Ethiopiafedluked130@gmail.com (F.K.S.); 2 Research Institute of Materials Chemistry, Chungnam National University, Daejeon 34134, Republic of Korea; ibs430@hotmail.com; 3 Department of Physics, Chungnam National University, Daejeon 34134, Republic of Korea; frendsamir@gmail.com; 4 Faculty of Biotechnology, Chemistry and Environmental Engineering, Phenikaa University, Hanoi 10000, Vietnam; trantan160288@gmail.com; 5 Institute of Fundamental Technological Research, Polish Academy of Sciences, 02-106 Warsaw, Poland; njemuwa.nwaji@zq-scnu.org

**Keywords:** electrodeposition, hydrogen evolution reactions, catalytic activity, Cu/MoS_2_ nanostructures

## Abstract

Molybdenum sulfide–oxide (MoS_2_, MS) emerges as the prime electrocatalyst candidate demonstrating hydrogen evolution reaction (HER) activity comparable to platinum (Pt). This study presents a facile electrochemical approach for fabricating a hybrid copper (Cu)/MoS_2_ (CMS) nanostructure thin-film electrocatalyst directly onto nickel foam (NF) without a binder or template. The synthesized CMS nanostructures were characterized utilizing energy-dispersive X-ray spectroscopy (EDS), scanning electron microscopy (SEM), X-ray diffraction (XRD), and electrochemical methods. The XRD result revealed that the Cu metal coating on MS results in the creation of an extremely crystalline CMS nanostructure with a well-defined interface. The hybrid nanostructures demonstrated higher hydrogen production, attributed to the synergistic interplay of morphology and electron distribution at the interface. The nanostructures displayed a significantly low overpotential of −149 mV at 10 mA cm^−2^ and a Tafel slope of 117 mV dec^−1^, indicating enhanced catalytic activity compared to pristine MoS_2_.This research underscores the significant enhancement of the HER performance and conductivity achieved by CMS, showcasing its potential applications in renewable energy.

## 1. Introduction

Energy and the environment are two major issues that are directly related to sustainable growth in society. Fossil fuels such as coal, oil, and natural gas have historically been thought of as the primary energy source. However, the combustion of fossil fuels releases detrimental greenhouse gases, notably carbon dioxide (CO_2_). Addressing this environmental challenge has prompted investigations into hydrogen as a viable alternative to fossil fuels [1,2]. Free hydrogen does not exist abundantly and is mainly present in compounds such as hydrocarbons and water [3]. Water electrolysis is an eco-friendly method for producing renewable hydrogen. This electrochemical process involves the anodic generation of O_2_, known as the “oxygen evolution reaction (OER)”, and the cathodic generation of H_2_, known as the “hydrogen evolution reaction (HER)” [4]. The hydrogen and oxygen evolution reactions happen only when a specific thermodynamic potential is reached: 0.00 V and 1.23 V for the HER and OER (vs. RHE), respectively [5]. However, in practical applications water electrolysis often necessitates voltages higher than the thermodynamic potential due to challenges such as high overpotential and sluggish kinetics [6]. To address this, research has been focusing primarily on the design of electrodes, electrocatalysts, and electrolytes to reduce overpotential and to obtain a better understanding of electrode kinetics [1].

During the HER, the improved electrocatalyst is a critical component that reduces electrode overpotential resulting in a high current density and enhancing the yield of this vital electrochemical process [7]. So far, electrocatalysts based on precious metals such as platinum and palladium have shown significant promise. Nonetheless, their high cost and unavailability have limited their widespread adoption [8]. As a result, much research has concentrated on producing electrocatalysts derived from abundant and non-precious minerals while keeping a remarkable electrocatalytic performance [9]. Earth-abundant transition-metal dichalcogenides (TMDs), which include two-dimensional (2D) layered materials, such as MoS_2_, MoSe_2_, WS_2_, WSe_2_, TaS_2_, and TiS_2_, with a general formula of MX_2_ (M = transition metal; X = chalcogen), have gained considerable attention in the field of electrocatalysis owing to their fascinating chemical and electronic features [10]. The MoS_2_ structure has piqued the interest of TMDs in the field of electrocatalysts owing to its S-edges with an ideal Gibbs free energy of hydrogen adsorption (GH*) of 0.06 eV, meaning that MoS_2′_s active sites are located along the edges with an energy level close to 0 eV [11].

The mechanism of the HER on different catalysts was studied in 2005 by Jens Nørskov’s research group using density functional theory (DFT) simulations. Their results showed that MoS_2_ nanoparticles’ Gibbs free energy (ΔG) for the HER is comparable to Pt’s, suggesting MoS_2′_s potential for the HER. Furthermore, subsequent experiments confirmed that the edges of MoS_2_ nanosheets are electrochemically active [10]. However, because of their strong van der Waals contacts across layers and high surface energy, MoS_2_ nanostructures tend to stack together, resulting in limited active sites and hindered electron transport. As a result, the low electrical conductivity and restricted active edges of MoS_2_ have limited its broader application [12]. To overcome these problems, the couplings of two or more material components to produce heterostructure catalysts with rich interfaces have recently drawn the attention of many researchers in this area [11].

A volcano plot of different catalyst metals depicted that the exchange current density of Cu is slightly lower than that of the noble Pt-group metals [13], which could mean that combining Cu with MoS_2_ could potentially improve the HER performance and conductivity [7]. The incorporation of Cu into the MoS_2_ catalyst has substantially increased the catalytic efficiency of the HER [14] and the nanostructure has also exhibited superior stability in acidic solutions and outstanding electrocatalytic activity with a Tafel slope of approximately 75.8 mV dec^−1^ [9]. In this work, an effective, controlled, simple, environmentally friendly, and cost-effective electrochemical deposition method has been employed for the synthesis of the nanostructures. Therefore, this study introduces a Cu/MoS_2_ nanostructure catalyst synthesized by a two-step electrodeposition method exhibiting superior stability in alkaline solutions and promising electrocatalytic activity for HERs.

## 2. Material and Method

### 2.1. Chemicals and Reagents

Nickel foam (NF) (purity > 99.99%; surface density 346 g/cm^2^; and thickness 1.6 mm) and fluorine-doped tin oxide (FTO) (resistivity 10 × 10^−5^ Ω/vm^2^) were obtained from MTI Korea. All chemicals, HCl (37%), Ammonium molbdate thiosulphate, ((NH_4_)_2_MoS_4_) (99.99%), KCl (99.95%), CuSO_4_·5H_2_O (97%), Ethanol (99.9%), and KOH ACS reagent ≥85% pellets, were purchased from Sigma-Aldrich. All chemicals were used without any further purification.

### 2.2. Synthesis of MoS_2_ and Cu/MoS_2_ Electrocatalysts

The MoS_2_ (MS) nanoparticles (NPs) were synthesized by a two-step electrochemical deposition method. The synthesis of MoS_2_ NPs on nickel foam (NF) was carried out according to the recently reported literature with a slight modification [15]. Prior to the deposition, the NF (20 mm length × 10 mm width × 1.6 mm thickness) was sonicated with 1 M HCl for 30 min to remove the oxide layer and the HCl-soaked NF was air-dried to remove the moisture. The electrochemical deposition was performed in a three-electrode cell using the NF (size 1 cm × 1 cm) as the working electrode, platinum wire as the counter electrode, and a Ag/AgCl electrode as the reference electrode. In the first step of the process, a voltage of −1.0 V was applied on an electrode immersed in a solution containing 7 mM ((NH_4_)_2_MoS_4_) and 0.1 M KCl for four different deposition times: 100 s, 200 s, 300 s, and 500 s coded as MS1, MS2, MS3, and MS5, respectively. The prepared MoS_2_ NPs were rinsed with water and ethanol and air-dried for 8 h. The Cu/MoS_2_ (CMS) nanostructures were deposited with slight modification of a previously reported method [16]. Then, 0.1 M of CuSO_4_·5H_2_O solution was deposited on MS electrode for four different deposition times, 60 s, 80 s, 100 s, and 120 s, at a voltage of −1.0 V, coded as 6-CMS, 8-CMS, 10-CMS, and 12-CMS, respectively. After the electrodeposition, the as-prepared CMS NPs were rinsed with ethanol and water followed by air-drying for 8 h.

### 2.3. Characterization of CMS Nanostructure

The crystal structure of the samples was analyzed using X-ray diffraction (XRD) using XRD-7000 X-RAY DIFFRACTOMETER, SHIMADZU Corporation (Kyoto, Japan). The morphologies of the synthesized nanomaterial and nanostructures were characterized using scanning electron microscopy (SEM) and transmission electron microscopy (TEM), FE-SEM S-4700, Hitachi, and TEM, JEM-2100F, JEOL Ltd., Tokyo, Japan. The elemental composition and distribution were investigated with energy dispersive X-ray spectroscopy (EDS, JEOL-2010) coupled with scanning electron microscopy (SEM, S-4700), and electrochemical measurements were carried out using Iviumstat electrochemical interface (Eindhoven, The Netherlands).

### 2.4. Electrochemical Measurements

All electrochemical measurements were performed in a standard three-electrode system using an Iviumstat (Eindhoven, The Netherlands) workstation at room temperature while using the as-obtained catalysts on the NF as the working electrode (WE), Pt wire as the counter electrode (CE), and Ag/AgCl electrode as the reference electrode (RE) in 1M KOH as the electrolyte solution. For the HER, the Linear Sweep voltammetry (LSVs) measurements were carried out at the potential range from −1.5 to 0 V vs. Ag/AgCl at a scan rate of 2 mV s^−1^. Electrochemical impedance spectroscopy (EIS) was measured in a frequency range of 1 × 10^5^ to 0.01 Hz at optimized potential of −1.16 V vs. Ag/AgCl. Cyclic voltammetry (CVs) measurements were carried out from −0.3 to −0.2 V vs. Ag/AgCl at scan rates of 20, 40, 60, 80, 100, and 120 mV s^−1^ to estimate the double-layer capacitances (Cdl) of the catalysts. Chronoamperometry curves were obtained with a constant current density of 10 mA cm^−2^. The potentials measured were converted to a reversible hydrogen electrode (RHE) using the following equation [16]:

*E_RHE_* = *E*°*_Ag/AgCl_* + 0.059 ∗ *pH* + *E_Ag/AgCl_*

**Figure 1:**
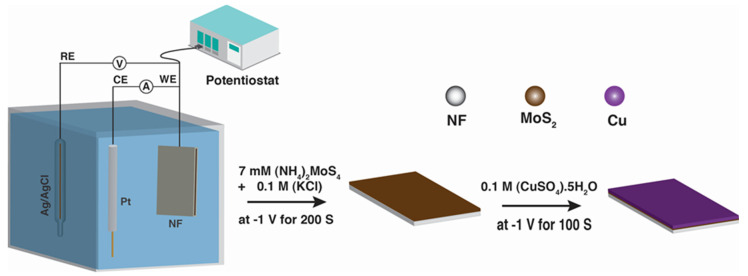
Schematic illustration of the fabrication of CMS.

The electrochemically active surface area (ECSA) was determined through the electrochemical double-layer capacitance (Cdl), assessed via straightforward cyclic voltammetry (CV) measurements conducted in a non-Faradaic region at various scan rates [17]. Figure 1 depicts schematic illustration of the fabrication of CMS nanostructures.

## 3. Result and Discussion

### 3.1. Fabrication and Characterization of CMS Nanostructures

The Cu/MoS_2_ (CMS) nanomaterials were synthesized through a two-step electrochemical deposition method, as illustrated in Figure 1. Initially, MoS_2_ (MS) nanoparticles (NPs) were electrodeposited onto nickel foam (NF) using a simple and cost-effective method at an applied potential of −1.0 V vs. Ag/AgCl for four distinct deposition times: 100 s, 200 s, 300 s, and 500 s, denoted as MS1, MS2, MS3, and MS5, respectively. The resulting MS was thoroughly cleaned using deionized (DI) water before being left to dry. Subsequently, 0.1 M CuSO_4_·5H_2_O solutions were electrodeposited onto the optimized MoS_2_ (MS) electrode for four different depositions time (60 s, 80 s, 100 s, and 120 s) at a voltage of −1.0 V, resulting in the synthesis of 6-CMS, 8-CMS, 10-CMS, and 12-CMS, respectively. The electrochemical deposition of MoS_2_ resulted in the NF turning from silvery to black. Subsequently, the black color transformed into reddish-brown upon Cu deposition on MS/NF, indicating the successful deposition of MoS_2_ and Cu/MoS_2_, respectively (see Figure S1).

**Figure 2:**
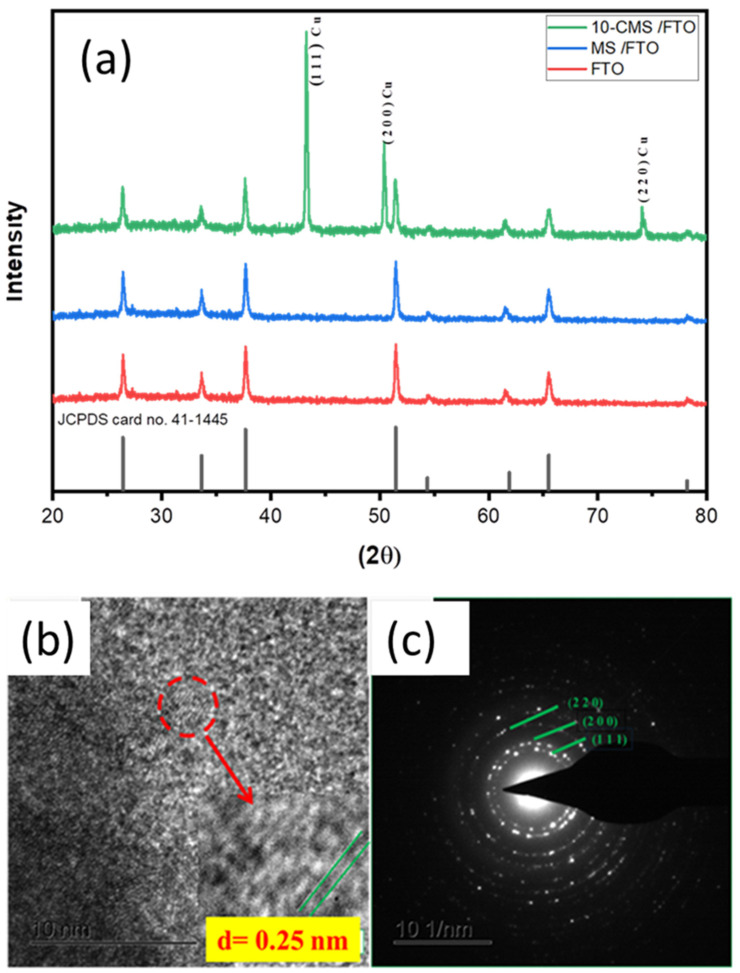
(**a**) XRD spectrum of bare FTO, MS/FTO, and 10-CMS/FTO NPs, (**b**) HRTEM images and (**c**) SAED images of 10-CMS nanostructures.

**Figure 3:**
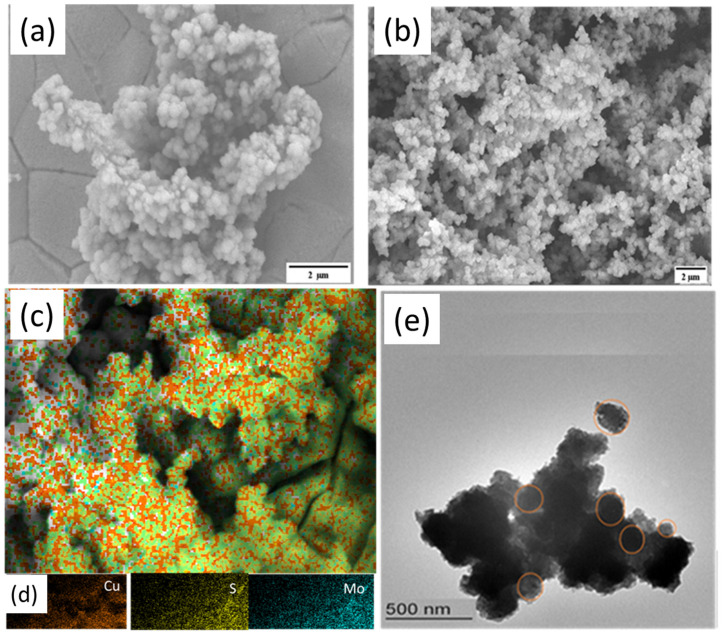
SEM images of (**a**) MS, (**b**) 10-CMS NPs, (**c**,**d**) the corresponding elemental mapping, and (**e**) TEM image of 10-CMS NPs.

The crystalline phases of the electrodeposited CMS/FTO films were characterized by XRD. Figure 2a reveals that there are no apparent XRD peaks of MS indicating amorphous MS films [16]. The presence of Cu has been confirmed by the peaks at 43.29, 50.4, and 74.13 with the crystal planes (1 1 1), (2 0 0), and (2 2 0), respectively (JCPDS card no. 04–0836) [7]. The FTO conductive glass substrate’s XRD pattern shows the characteristic diffraction peaks at 27°, 35°, 39°, 52°, 55°, 62°, 67°, and 79° which are attributed to the tetragonal SnO_2_ (JCPDS card no. 41-1445) of the CMS/FTO film (JCPDS No. 01-077-0452). The CMS/FTO has been used instead of the CMS /NF film for XRD analysis due to the high background signal of Ni generated by NF. High-Resolution Transmission Electron Microscopy (HRTEM) images in Figure 2b show an interplanar distance of 0.25 nm compatible with the interplanar distance of the the Cu (111) crystallographic plane, which is also in agreement with the XRD results (Figure 2a). Figure 3c shows the selected area electron diffraction (SAED) pattern of CMS NPs displaying a distinct ring pattern. This regular ring pattern is indicative of the existence of crystalline Cu NPs. The lattice fringes characterized by spacing values of 0.25 nm, 0.2271 nm, and 0.1261 nm correspond to the (1 1 1), (2 0 0), and (2 2 0) planes of Cu, respectively, aligning remarkably well with the XRD pattern [7].

As depicted in Figure 3a, the SEM analysis reveals that MS exhibits spherical particle shapes with a particle size of 246. The large size of the particles results in a small surface area to volume ratio and limited active sites of the material that negatively affects its catalytic activity. On the other hand, the electrodeposition of Cu on MS resulted in spherically shaped and uniformly distributed particles with a particle size of 154 nm (Figure 3b). This indicates that Cu has played a crucial role in dispersing the MS particles on the substrate, leading to an increase in the surface area to volume ratio and active sites available for catalysis which could significantly improve the catalytic activity of CMS.

The elemental mapping data provide additional information about the distribution of elements on the sample surface. Accordingly, the map shows the uniform distribution of Cu on the surface, appearing as bright spots, Figure 3c,d. Moreover, the Mo and S elements appear to be more evenly distributed across the surface, suggesting that the MoS_2_ layer is still present and intact despite the presence of Cu. To further study the detailed microstructure of the as-prepared products, CMS was scratched from the NF and examined by TEM. Figure 3e shows the TEM micrographs of 10-CMS exhibiting very finely grained Cu NPs with spherical shapes which agrees with the result obtained from the SEM image (Figure 3b). The EDS analysis shown in Figure S2 confirms the presence of Mo, Cu, S, O, and Ni (from NF) in the sample. Furthermore, the air contact causes CMS’s surface to be oxidized which may be the cause of the oxygen peaks [9].

### 3.2. HER Catalytic Performance

The as-synthesized materials (MS, CMS) were utilized directly as HER working electrodes, platinum wire served as a counter electrode, and Ag/AgCl was used as the reference electrodes in a three-electrode system to analyze the electrochemical performance of the materials. The electrochemical behavior for the HER of all the materials was analyzed in alkaline media (1M KOH at a scan rate of 2.0 mV/s). In order to optimize the catalytic activity of MoS_2_ in the HER, the deposition time of MoS_2_ was varied at a constant concentration (7 mM) and potential (−1 V). Considering the results of the linear sweep voltammetry (LSV) and cyclic voltammetry (CV) shown in Figure S3a,b, the MS2 exhibited a better HER performance compared to the MS1, MS3, and MS5 NPs. The CV and LSV results showed that the MS2 had a larger area under the HER peak and a lower overpotential than MS3 and MS5. The larger area under the HER peak indicates a higher level of catalytic activity towards the HER, whereas the lower overpotential indicates a more efficient HER process [18]. The decreased HER performance of MS3 and MS5 compared to the MS2 can be attributed to the mass loading on the NF surface. Due to the high mass loading of the catalyst on the electrode, not every surface of the catalyst can actively engage in electrochemical reactions. This limitation arises because not all areas of the catalyst’s surface can be adequately wetted by the electrolyte, particularly in regions distanced from the interface between the electrolyte and the surface of the casted catalyst layer on the electrode [19]. Furthermore, the amount of Cu deposited in the host material of MoS_2_ materials has been tuned. Accordingly, as shown in Figure S4, large CV curve areas have been obtained for all MS electrodes coated with Cu (6-CMS, 8-CMS, 10-CMS, and 12-CMS) as compared with MS-only electrodes. Among all CMS electrodes, 10-CMS has shown the largest area resulting in an increase in the electro-catalytic activity for the HER. The inclusion of Cu in MS boosted the conductivity of the nanostructure as Cu NPs can act as electron donors providing electrons to the MS. This transfer of electrons can promote the catalytic activity of both materials, leading to a greater overall catalytic activity than either material alone. Furthermore, the resulting hybrid has a large number of active HER catalytic sites due to the number of accessible edges resulting from the small size and irregular shape of MS. Through its synergistic effect with nano-sized MS, the copper coating not only increased the catalyst’s electrical conductivity, but also increased its catalytic activity [7]. However, for 12-CMS, the area of the CV curve diminished due to the fact that the overloaded Cu NPs restricted the hydrogen/electron transfer process, negating the beneficial effect of mechanical strain on the MoS_2_ (MS) NPs’ electrochemical activity in the direction of the HER. High charge transfer resistance and inadequate in-plane alignment are the causes of this effect [20].

**Figure 4:**
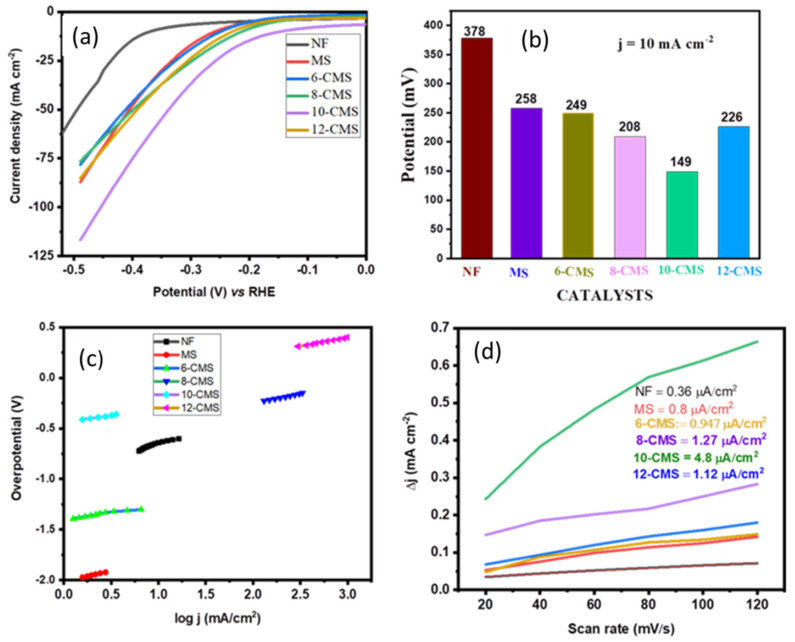
(**a**) LSV polarization curves at a scan rate of 2 mV s^−1^, (**b**) overpotential comparison, (**c**) Tafel curve of various CMS NSs carried out under a constant current density of 10 mA cm^−2^, and (**d**) capacitive current differences (Δ*j* = *janode* − *jcathode*) at −0.25 V vs. Ag/AgCl.

Figure 4a shows LSV curves of NF, MS, 6-CMS, 8-CMS, 10-CMS, and 12-CMS. To achieve a current density of 10 mA cm^−2^, nickel foam (NF) necessitates a voltage of −0.378 V, while MoS_2_ (MS) requires −0.258 V, and 10-CMS requires only −0.149 V vs. the reversible hydrogen electrode (RHE). This represents a reduction of approximately 1.736 times compared to the MS nanostructure at 10 mA cm^−2^. After Cu and MS were successfully combined, there was a noticeable cathodic change in the onset potential, which suggests that a Cu and MS nanostructure combination significantly enhanced the electrocatalytic performance of the MS nanostructure. Additionally, as illustrated in Figure 4b, a clear comparison of overpotential is presented, revealing that 10-CMS exhibits notably small overpotential. Furthermore, the high hydrogen evolution reaction activity of 10-CMS is comparable with many previously reported MS_-_based CMS/NF electrocatalysts, as shown in Table 1.

In electrochemistry, the reaction kinetics of electrochemical reactions are described by a Tafel slope. The sensitive response of the current to overpotential can be illustrated by plotting the logarithm of the current density (log(j)) against η [17]. Faster reaction kinetics are indicated by a lower Tafel slope value, while slower reaction kinetics are indicated by a greater Tafel slope value and this is typically calculated using the Tafel equation: η = blog J + a [12]. The rate-limiting step of the HER can be inferred from the Tafel slope, which indicates different reaction mechanisms via Volmer–Heyrovsky reactions between 40 and 120 mV dec^−1^ or Volmer–Tafel reactions between 29 and 39 mV dec^−1^ [5]. As shown in Figure 4c, Tafel slopes of 10-CMS are around 117 mV dec^−1^ in the 40–120 mV dec^−1^ range suggesting that the Volmer–Heyrovsky mechanism might have been followed by the HER process on the electrode surface in conjunction with the reaction mechanism.

H_2_O + e^−^ + ∗ → H* + OH^−^  (Volmer reaction)

H* + H_2_O + e^−^ → H_2_ + OH^−^ + ∗  (Heyrovsky reaction)

where ∗ is active site. This suggests that the barrier of the electrochemical water dissociation controls the first electron transfer step (Volmer step). CV is a helpful method to calculate the electrochemically active surface area [12]. The CV measurements were carried out in 1 M KOH at different scan rates between 20 and 120 mV s^−1^, within the potential window of −0.3 to −0.2 V vs. Ag/AgCl, as shown in Figure S5. The values of the double-layer capacitance (C_dl_) for NF, MS, 6-CMS, 8-CMS, 12-CMS, and 10-CMS are determined by calculating the slope of the current density vs. the scan rate. The values were 0.36, 0.8, 0.96, 1.12, 1.26, and 4.8 µF, respectively, as shown in Figure 4d. The C_dl_ values are normalized by CS (specific capacitance) to find out the ECSA. The specific capacitance value for a standard with 1 cm^2^ of real surface area can be used to further convert the C_dl_ into ECSA [25]. A flat surface’s specific capacitance typically ranges from 0.02 to 0.06 mF cm^−2^. Based on commonly reported values, a specific capacitance of 0.04 mF cm^−2^ was used for this work, which is a typical value for a metal electrode in an aqueous 1M KOH solution [26]. The calculated ECSA values for NF, MS, 6-CMS, 8-CMS, 12-CMS, and 10-CMS are 0.009, 0.02, 0.024 0.028, 0.0315, and 0.121 cm^2^, respectively. Additionally, 10-CMS exhibits stronger electrocatalytic activity than MS, as evidenced by the improved double-layer capacitance and ECSA. A further measure used to analyze an electrocatalyst’s electrocatalytic performance is the roughness factor (R_f_). Values for the R_f_ (active site per unit surface area) are calculated by normalizing ECSA using geometric surface areas (1 cm^2^) [5]. The highest R_f_ value of 0.121 was determined for 10-CMS.

**Figure 5:**
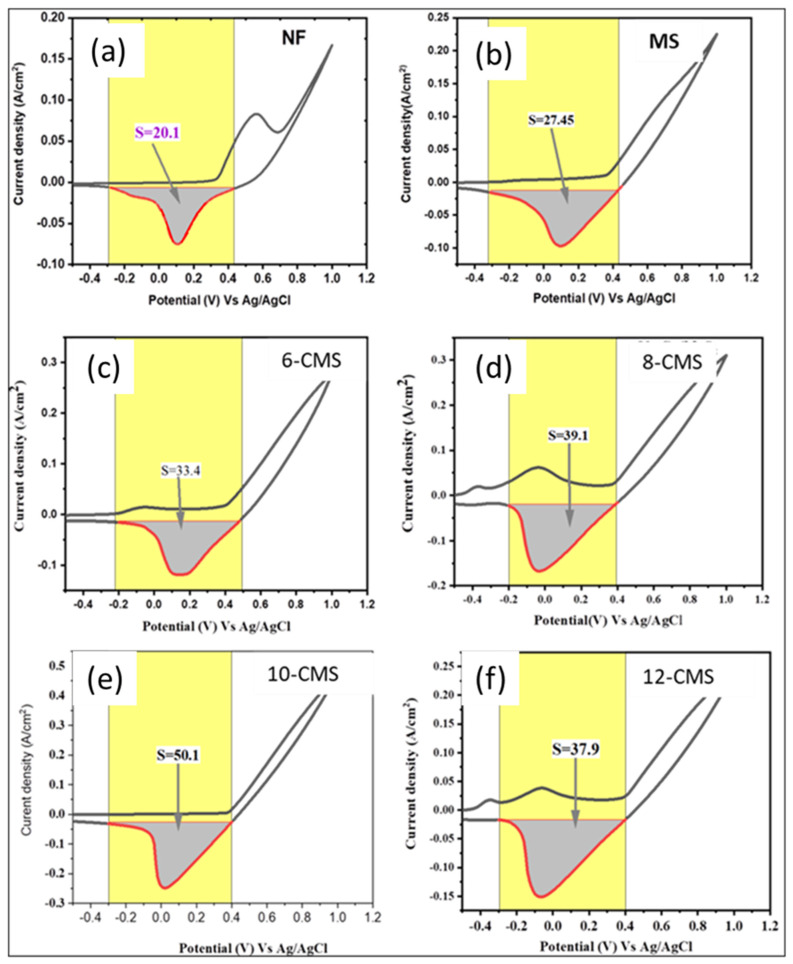
The reduction region of CV curves of (**a**) NF, (**b**) MS, (**c**) 6-CMS, (**d**) 8-CMS, (**e**) 10-CMS, and (**f**) 12-CMS measured at a scan rate of 50 mV s^−1^.

An additional quantitative metric for analyzing an electrocatalyst at a specified overpotential value is the turnover frequency (TOF). The number of moles of O_2_/H_2_ evolved in a unit of time is known as the catalyst’s TOF [27]. The following formula was used to determine the TOF for the HER.

$$TOF=\frac{|J|\;A}{2F\cdot n}$$

where *A* is the geometric area of the working electrode (1 cm^2^), *F* is the Faraday constant (96,485 C·mol^−1^), and n is the number of active sites. In the LSV measurement, |*J*| (A·cm^−2^) is the current density at a fixed voltage. By integrating the acquired CV curve, one may determine the charge *Q*, which is proportional to the number of active sites (*n*). Thus, the following equation was used to determine the active sites:

$$n=\frac{Q}{2F}=\frac{I\cdot t}{2F}=\frac{I\cdot V/u}{2F}=\frac{S}{2F\cdot v}$$

where *S* is the integrated effective reduction area, *I* is the current (A), *V* is the voltage (V), and *v* is the scan rate [28]. Using the reduction area of the CV curves recorded at 50 mVs^−1^, as shown in Figure 5, the surface’s active site concentrations were calculated and found to be 2.1 × 10^−6^ atoms cm^−2^, 2.8 × 10^−6^ atoms cm^−2^, 3.4 × 10^−6^ atoms cm^−2^, 3.95 × 10^−6^ atoms cm^−2^, 3.83 × 10^−6^, and 5.2 × 10^−6^ atoms cm^–2^ active components for NF, MS, 6-CMS, 8-CMS, 12-CMS, and 10-CMS, respectively. The addition of more copper nanomaterial (Cu) to molybdenum disulfide (MoS_2_) results in a slight increase in the concentrations of the number of active sites. The calculated TOF for 10-CMS was found to be 22 s^–1^ at 240 mV, which is higher than those of NF (12.5 s^−1^), MS (14 s^−1^), 6-CMS (16 s^−1^), 8-CMS (19 s^−1^), and 12-CMS (18.4 s^−1^), indicating the superior intrinsic HER activity of 10-CMS.

**Figure 6:**
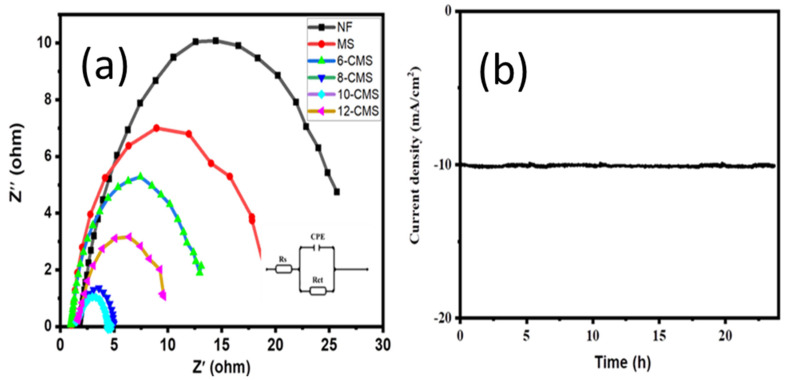
(**a**) Nyquist plots of CMS carried out under a constant −149 mV vs. RHE and equivalent electrical circuit inset. (**b**) Long-term stability test of 10-CMS carried out under a constant applied potential −149 mV vs. RHE.

Electrochemical impedance spectroscopy (EIS) was utilized to investigate the kinetics of the electrode at the electrolyte/electrode interface [29]. The charge transfer between the electrolyte and the electrocatalyst is a crucial factor that affects the efficiency of the HER process. The EIS analysis aids in identifying both the solution resistance and charge transfer resistance which arises from charge transport at the interface between the electrocatalyst and electrolyte. A lower value indicates a faster rate of charge transfer [12]. The results depicted in Figure 6a indicate that 10-CMS has a smaller R_ct_ value of 2.704 Ω compared to MS (16.9 Ω) and bare NF (25.7 Ω), indicating a more rapid charge transfer rate that is beneficial for an efficient HER process. This suggests that 10-CMS is a more effective electrocatalyst for the HER process compared to the other materials. The stability of an electrode can be evaluated by performing chronoamperometry, which involves applying a constant potential to the electrode and measuring the resulting current over time. In this case, the 10-CMS electrode produced a constant current density of 10 mA/cm^2^ for a continuous 24 h period, as shown in Figure 6b, suggesting that the electrode is stable and has good electrocatalytic activity for the reaction being studied.

## 4. Conclusions

In this study, the electric-field-assisted synthesis method has been employed for synthesizing highly dispersed CMS catalysts with improved catalytic activity for the HER. The 10-CMS catalysts exhibited the highest hydrogen production due to the synergistic effect of the morphology and electron distribution across the interface, exhibiting a low overpotential of −149 mV at 10 mA cm^−2^ and a Tafel slope of 117 mV dec^−1^, showing superior catalytic activity than bare MoS_2_. In addition, the Cu deposited on the MoS_2_ surface served as active sites for the HER and improved the electrical conductivity of the MoS_2_ substrate by facilitating electron transfer during the HER. Hence, the findings of this study highlight the potential of the electric-field-assisted synthesis method and modifications to the MoS_2_ substrate in improving the performance of MS-based catalysts in HER catalysis. Furthermore, this study contributes to the development of efficient and cost-effective catalysts for the HER and advances the progress towards a sustainable hydrogen economy.

## Figures and Tables

**Table 1:** Comparison of the electrocatalytic activity of the as-prepared CMS (current density of 10 mA cm^−2^) nanomaterial toward HER with previously reported MoS_2_-based catalysts.

Prepared Sample	Electrolyte	Over-Potential η (mV)	Synthesis Method	Ref
10-CMS	KOH	149	Electrodeposition Method	This work
MWCNTs@Cu@MoS_2_	H_2_SO_4_	146	Solvothermal Method	[21]
MoS_2_/Cu	KOH	160	Etching Method	[9]
Ag-Ag_2_S/MoS_2_	H_2_SO_4_	150	Microwave-assisted technique	[12]
Etched MoS_2_	H_2_SO_4_	316	One-step microwave-assisted method	[7]
MoS_2_ Quantum Dot	H_2_SO_4_	190	Hydrothermal Method	[22]
MoS_2_NTs array	H_2_SO_4_	296	Electrophoretic Deposition	[8]
Cu-MoS_2_-SV	H_2_SO_4_	197	Chemical Etching Method	[23]
Cu_9_S_5_@MoS_2_	KOH	146	Chemical Vapor Deposition (CVD)	[24]

## Data Availability

The data that support the findings of this study are available from the corresponding authors upon reasonable request.

## Supplementary Materials

The following supporting information can be downloaded at: https://www.mdpi.com/article/10.3390/mi15040495/s1, Figure S1: Digital photographs of (a) bare NF, (b) MS, and (c) CMS NSs; Figure S2. SEM-EDS spectrum of 10-CMS; Figure S3. (a) CV curves recorded at a scan rate of 50 mV s^−1^, Electrochemical performance of various MS NSs electrodes carried out under a constant current density of 10 mA cm^−2^ in 1 M KOH aqueous solution. (b) HER polarization curves; Figure S4: CV curves of NF, MoS_2_, and CMS s recorded at a scan rate of 50 mV s^−1^. Figure S5: Double layer capacitance measurements with scan rates at 20, 40, 60, 80, 100 and 120 mV s^−1^ in the region of –0.3 to –0.2 V vs. Ag/AgCl for CMS/NF.

## References

[1] Samantara A.K., Ratha S. (2019). Mechanism and Key Parameters for Catalyst Evaluation. Metal Oxides/Chalcogenides and Composites. Springer Briefs in Materials.

[2] Alonso-Vante N., Roldán C.A.C., Huerta R.d.G.G., Sánchez G.R., Robledo A.M. (2019). Fundamentals of Electrocatalyst Materials and Interfacial Characterization.

[3] Tiwari A.P., Novak T.G., Bu X., Ho J.C., Jeon S. (2018). Layered Ternary and Quaternary Transition Metal Chalcogenide Based Catalysts for Water Splitting. Catalysts.

[4] Herbaut M., Siaj M., Claverie J.P. (2021). Nanomaterials-Based Water Splitting: How Far Are We from a Sustainable Solution?. ACS Appl. Nano Mater..

[5] Samantara A.K., Ratha S. (2018). Materials Development for Active/Passive Components of a Supercapacitor: Background, Present Status and Future Perspective.

[6] Bhat K.S., Nagaraja H.S. (2021). Recent trends and insights in nickel chalcogenide nanostructures for water-splitting reactions. Mater. Res. Innov..

[7] Cao J., Zhou J., Zhang Y., Liu X. (2017). A facile one-step fabrication of a novel Cu/MoS2 nano-assembled structure for enhanced hydrogen evolution reaction performance. RSC Adv..

[8] Pataniya P.M., Sumesh C.K. (2022). MoS2 nanosheets on Cu-foil for rapid electrocatalytic hydrogen evolution reaction. J. Electroanal. Chem..

[9] Ilyas T., Raziq F., Ali S., Zada A., Ilyas N., Shaha R., Wang Y., Qiao L. (2021). Facile synthesis of MoS2/Cu as trifunctional catalyst for electrochemical overall water splitting and photocatalytic CO_2_ conversion. Mater. Des..

[10] Wang F., Shifa T.A., Zhan X., Huang Y., Liu K., Cheng Z., Jiang C., He J. (2015). Recent advances in transition-metal dichalcogenide based nanomaterials for water splitting. Nanoscale.

[11] Sun J., Meng X. (2021). Modulating the Electronic Properties of MoS2Nanosheets for Electrochemical Hydrogen Production: A Review. ACS Appl. Nano Mater..

[12] Sharma M.D., Mahala C., Modak B., Pande S., Basu M. (2021). Doping of MoS2by ‘cu’ and ‘v’: An Efficient Strategy for the Enhancement of Hydrogen Evolution Activity. Langmuir.

[13] Li F., Zhang L., Li J., Lin X., Li X., Fang Y., Huang J., Li W., Tian M., Jin J. (2015). Synthesis of Cu e MoS2/rGO hybrid as non-noble metal electrocatalysts for the hydrogen evolution reaction. J. Power Sources.

[14] Gudal C.C., Pan U.N., Paudel D.R., Kandel M.R., Kim N.H., Lee J.H. (2022). Bifunctional P-Intercalated and Doped Metallic (1T)-Copper Molybdenum Sul fi de Ultrathin 2D-Nanosheets with Enlarged Interlayers for Efficient Overall Water Splitting. ACS Appl. Mater. Interfaces.

[15] Quy V.H.V., Vijayakumar E., Ho P., Park J.-H., Rajesh J.A., Kwon J., Chae J., Kim J.-H., Kang S.-H., Ahn K.-S. (2018). Electrodeposited MoS2 as electrocatalytic counter electrode for quantum dot- and dye-sensitized solar cells. Electrochim. Acta.

[16] Gicha B.B., Tufa L.T., Kang S., Goddati M., Bekele E.T., Lee J. (2021). Transition metal-based 2D layered double hydroxide nanosheets: Design strategies and applications in oxygen evolution reaction. Nanomaterials.

[17] Yu L., Zhou H., Sun J., Qin F., Luo D., Xie L., Yu F., Bao J., Li Y., Yu Y. (2017). Hierarchical Cu@CoFe layered double hydroxide core-shell nanoarchitectures as bifunctional electrocatalysts for efficient overall water splitting. Nano Energy.

[18] Choudhury B.J., Roy K., Moholkar V.S. (2021). Improvement of Supercapacitor Performance through Enhanced Interfacial Interactions Induced by Sonication. Ind. Eng. Chem. Res..

[19] Yu L., Sun S., Li H., Xu Z.J. (2021). Effects of catalyst mass loading on electrocatalytic activity: An example of oxygen evolution reaction. Fundam. Res..

[20] Lee J.H., Jang W.S., Han S.W., Baik H.K. (2014). Efficient hydrogen evolution by mechanically strained MoS2 nanosheets. Langmuir.

[21] Li F., Li J., Lin X., Li X., Fang Y., Jiao L., An X., Fu Y., Jin J., Li R. (2015). Designed synthesis of multi-walled carbon nanotubes@Cu@MoS2 hybrid as advanced electrocatalyst for highly efficient hydrogen evolution reaction. J. Power Sources.

[22] Han D., Luo Z., Li Y., Gao N., Ge J., Liu C., Xing W. (2020). Synergistic engineering of MoS2 via dual-metal doping strategy towards hydrogen evolution reaction. Appl. Surf. Sci..

[23] Liu Y., Guan S., Du X., Chen Y., Yang Y., Chen K., Zheng Z., Wang X., Shen X., Hu C. (2023). S-Vacancy Defect and Transition-Metal Atom Doping to Trigger Hydrogen Evolution of Two-Dimensional MoS 2. Energy Fuels.

[24] Zhang Z., Zhu H., Hao J., Lu S., Duan F., Xu F., Du M. (2021). One-dimensional, space-confined, solid-phase growth of the Cu9S5@MoS2 core–shell heterostructure for electrocatalytic hydrogen evolution. J. Colloid. Interface Sci..

[25] Connor P., Schuch J., Kaiser B., Jaegermann W. (2020). The Determination of Electrochemical Active Surface Area and Specific Capacity Revisited for the System MnOx as an Oxygen Evolution Catalyst. Z. Fur Phys. Chem..

[26] Duan J., Chen S., Vasileff A., Qiao S.Z. (2016). Anion and Cation Modulation in Metal Compounds for Bifunctional Overall Water Splitting. ACS Nano.

[27] Anantharaj S., Ede S.R., Sakthikumar K., Karthick K., Mishra S., Kundu S. (2016). Recent Trends and Perspectives in Electrochemical Water Splitting with an Emphasis on Sulfide, Selenide, and Phosphide Catalysts of Fe, Co, and Ni: A Review. ACS Catal..

[28] Tong J., Li Y., Bo L., Li W., Li T., Zhang Q., Kong D., Wang H., Li C. (2019). CoP/N-Doped Carbon Hollow Spheres Anchored on Electrospinning Core-Shell N-Doped Carbon Nanofibers as Efficient Electrocatalysts for Water Splitting. ACS Sustain. Chem. Eng..

[29] Aguedo J., Lorencova L., Barath M., Farkas P., Tkac J. (2020). Electrochemical impedance spectroscopy on 2D nanomaterial mxene modified interfaces: Application as a characterization and transducing tool. Chemosensors.

