# Supplementary material for: Electric-Field-Assisted Synthesis of Cu/MoS2 Nanostructures for Efficient Hydrogen Evolution Reaction
Source: Micromachines (Basel). 2024 Apr 3;15(4):495. doi: 10.3390/mi15040495 (PMC11052344; doi:10.3390/mi15040495)
Supplement: Supplementary file 1 [file micromachines-15-00495-s001.zip › micromachines-2922189-supplementary.pdf]

Supporting Information

Electric-Field-Assisted Synthesis of Cu/MoS_2 Nanostructures for Efficient Hydrogen Evolution Reaction

Surra Yonas[a], Birhanu Bayissa Gicha[b], Samir Adhikari[c], Fedlu Kedir Sabir[a], Van Tan Tran[e], Njemuwa Nwaji[f], Bedasa Abdisa Gonfa[a,*], Lemma Teshome Tufa[a,b,*]

[a]Department of Applied Chemistry, Adama Science and Technology University, P.O.Box 1888, Adama, Ethiopia

[b]Research Institute of Materials Chemistry, Chungnam National University, Daejeon 34134, Republic of Korea

[c]Department of Physics, Chungnam National University, Daejeon, 34134, Republic of Korea

[d]Faculty of Biotechnology, Chemistry and Environmental Engineering, Phenikaa University, Hanoi 10000, Viet Nam

[f]Institute of Fundamental Technological Research, Polish Academy of Sciences, 02-106, Warsaw, Poland

Corresponding authors: BAG, bedassa.abdissa@astu.edu.et; LTT, lemmat2003@yahoo.com

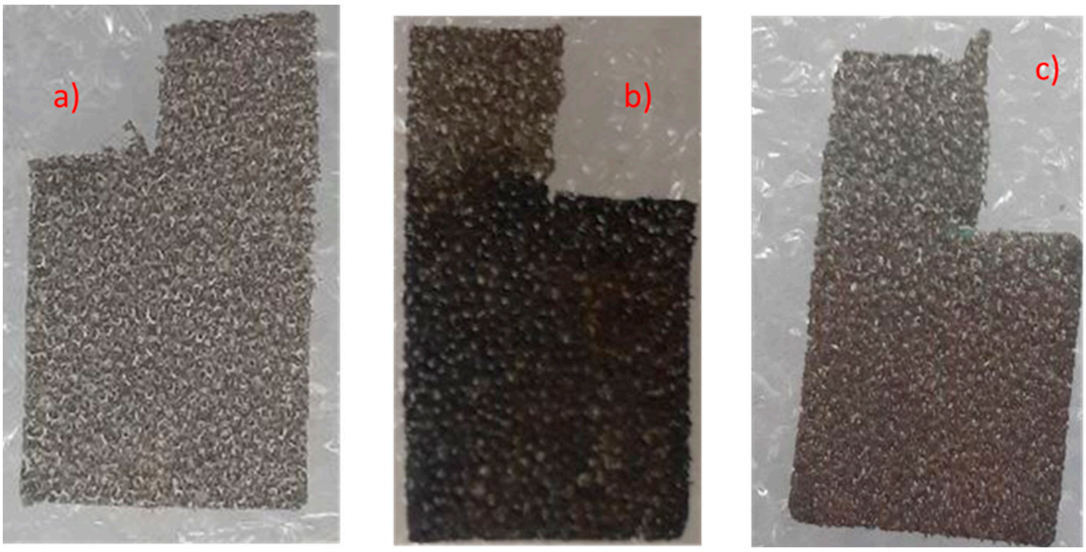

Figure S1. Digital photographs of (a) bare NF, (b) MS, and (c) CMS NSs

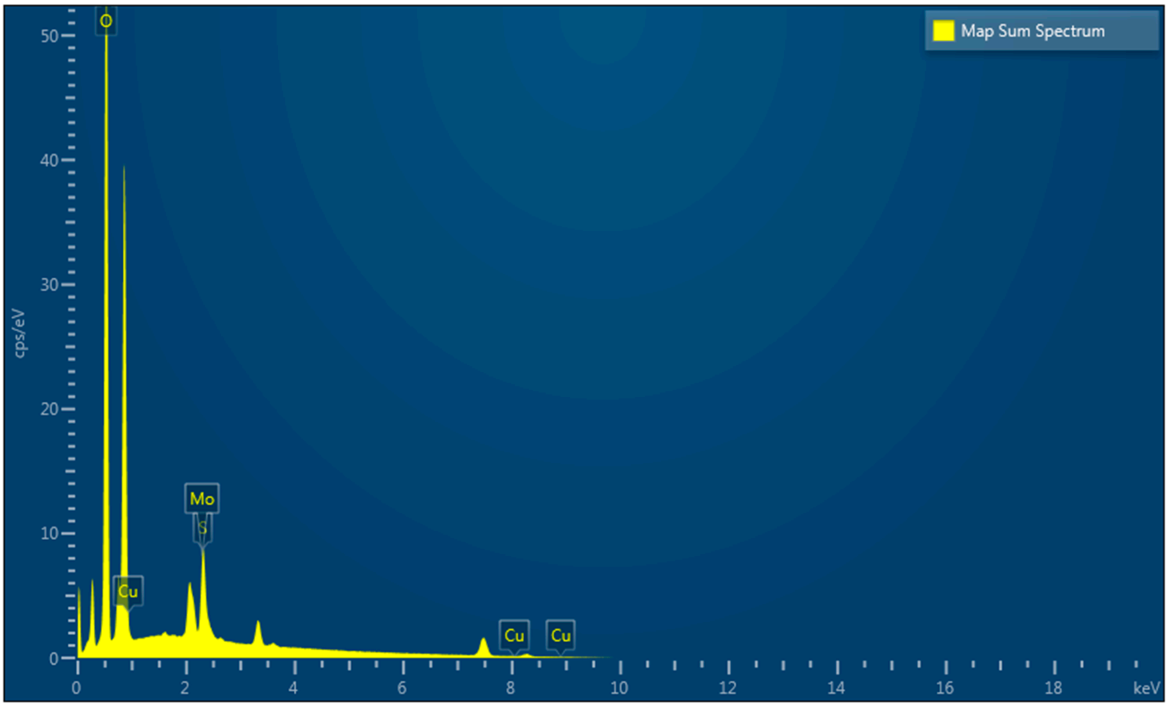

Figure S2. SEM-EDS spectrum of 10-CMS

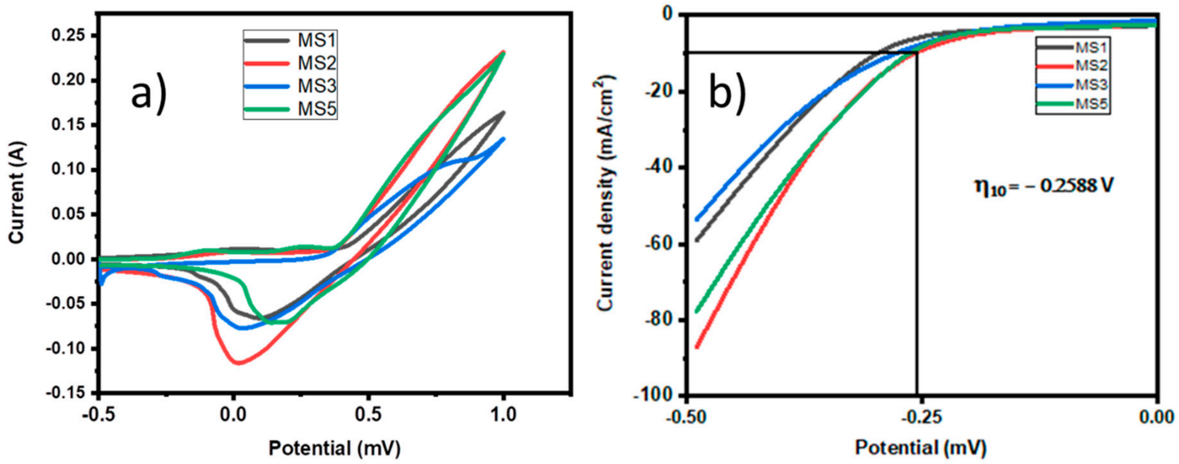

Figure S3. (a) CV curves recorded at a scan rate of 50 mV s^{-1} , Electrochemical performance of various MS NSs electrodes carried out under a constant current density of 10 mA cm^{-2} in 1 M KOH aqueous solution. (b) HER polarization curves

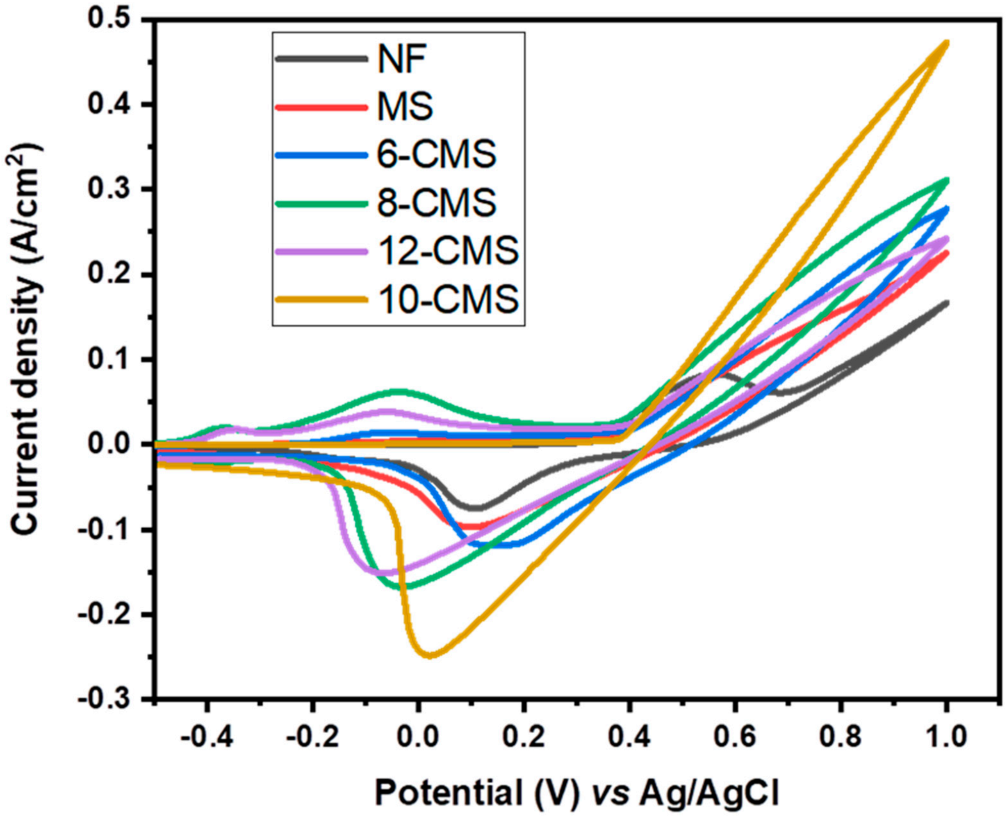

Figure S4 : CV curves of NF, MoS_2, and CMS s recorded at a scan rate of 50 mV s^{-1}.

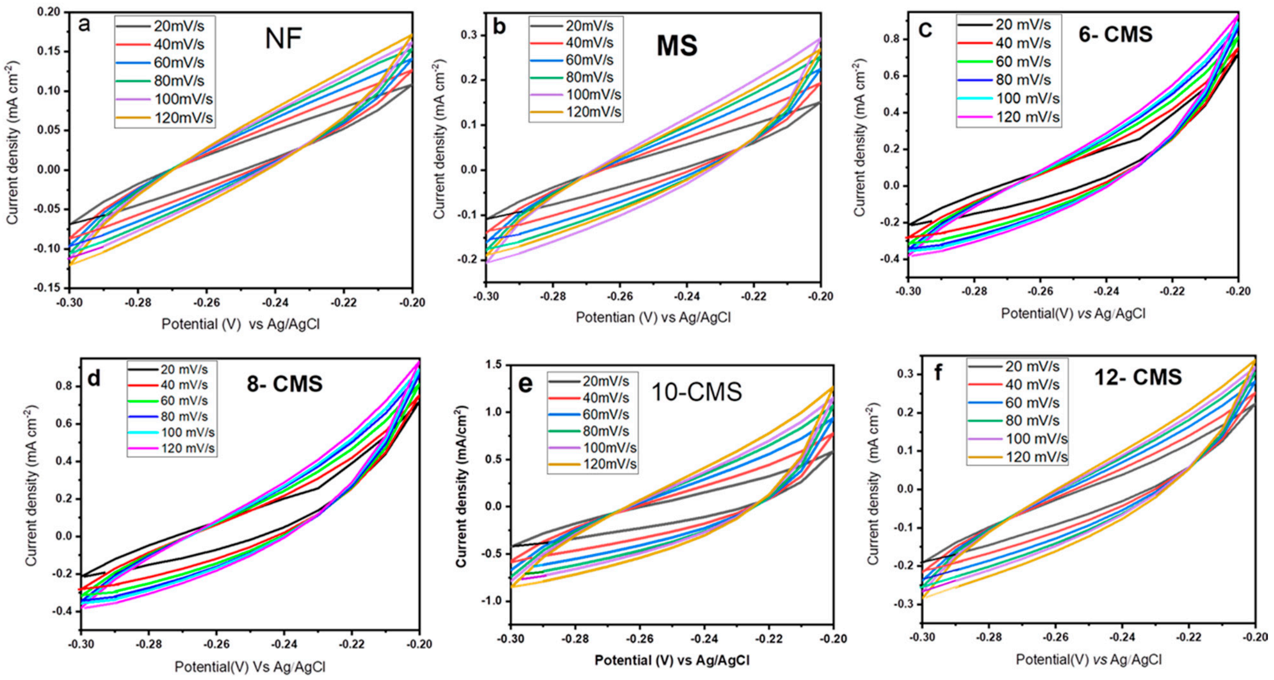

Figure S5: Double layer capacitance measurements with scan rates at 20, 40, 60, 80, 100 and 120 mV/s in the region of – 0.3 to –0.2 V vs. Ag/AgCl for CMS/NF.
